# Clinical epidemiology of venous thromboembolic disease: An institutional registry

**DOI:** 10.3389/fcvm.2022.928094

**Published:** 2022-07-22

**Authors:** Mohammed AlSheef, Fouad Taiwilaa Alshammari, Mashel khalid Alhawish, Abduljabar Ghazi Alghamdi, Abdullah Fahad Alqudhybi, Amani Abu-Shaheen

**Affiliations:** ^1^King Fahad Medical City, Riyadh, Saudi Arabia; ^2^Research Center, King Fahad Medical City, Riyadh, Saudi Arabia

**Keywords:** venous thromboembolism, PE and DVT, epidemiology, VTE, unprovoked VTE, provoked VTE

## Abstract

**Introduction:**

Venous thromboembolism (VTE) is a major health concern, with an annual incidence of ~1 in 1,000. The epidemiology of VTE in Saudi Arabia has not been adequately described yet. Therefore, this study aimed to assess the clinical characteristics, risk factors, diagnostic methods, management, and clinical outcomes of patients with VTE.

**Methods:**

This study was based on a VTE registry created over ten years at King Fahad Medical City (KFMC) in Riyadh, Saudi Arabia. All adult inpatients and outpatients referred to the thrombosis unit of the KFMC with clinically suspected VTE including pulmonary embolism (PE) and deep vein thrombosis (DVT) were enrolled. Data were collected using a standardized case report form, which included demographic and clinical characteristics, risk factors, diagnostic methods, management, and outcomes.

**Results:**

A total of 1,008 patients were recruited. Most patients were women (73.2%), and more than half of all patients had unprovoked VTE (58%). Among the provoked cases, the most frequent cause was surgery (29.8%), followed by hospitalization (24.2%). There was a significant statistical association between provoked status and sex, family history of VTE, smoking, recent hospitalization within 3 months for a medical condition, the site of VTE, and underlying peripheral vascular disease and varicose veins (all *p* < 0.05). The majority (88.3%) of patients with deep vein thrombosis was hospitalized for ≤3 days (*n* = 433, 79.9%), while fewer than half of the patients with PE needed hospitalization (45.3%). Thrombolytic therapy was administered to 14.1% (*n* = 142) of patients, and catheter-directed thrombolysis was performed in 1.0% (*n* = 10) of patients. The odds of mortality for provoked VTE were 3.20 times higher than those of unprovoked VTE [2.12–4.83; *p*-value < 0.001].

**Conclusion:**

Unprovoked VTE was more common than provoked VTE in the Saudi Arabian cohort, implying hereditary predisposition. Furthermore, male sex, family history of VTE, prior history of VTE, type of VTE, underlying obesity, history of trauma, surgery, hospitalization, pregnancy, and 3–6 months of anticoagulation therapy were the most critical risk factors for VTE recurrence. The treatment patterns and clinical results were comparable to those reported in the literature.

## Introduction

Venous thromboembolism (VTE) including both pulmonary embolism (PE) and deep vein thrombosis (DVT) is considered a major health problem, with reported annual incidence of ~1 in 1,000 (1). There are several risk factors associated with thrombosis, including but not limited to: older age (2), obesity (3), history of thrombosis (4), surgery (4), hospitalization (2), varicose vein (4), thrombophilia (5), oral contraceptive (2), and pregnancy (6).

Furthermore, it has been reported that the variations in the genetic background of rare diseases vary across different populations and ethnicities, for example, Factor V Leiden is an important risk factor for VTE found in nearly 4.4% of the European population (7). VTE is also associated with environmental risk factors that are classified as provoked and unprovoked (2). Provoked risk factors may be transient (e.g., a recent surgery) or persistent and progressive (example due to metastatic cancer) (1). If patients had neither an important transient nor a persistent provoking risk factor for thrombosis, it is referred to as having “unprovoked” VTE (8). Acute VTE should not be viewed in isolation, and physicians ought to investigate other contributing factors, thus ensuring proper management and reducing the probability of recurrence (9, 10). A large clinical trial can potentially change the local screening and diagnostic processes in patients with suspected hypercoagulation (11). Accordingly, it is necessary to understand any risk factors or diseases that could be associated with VTE and design strategies to prevent its occurrence, especially in patients with co-morbidities or autoimmune diseases, instead of relying solely on international treatment standards; thus, each patient should be treated based medical investigations (10).

However, the impact of these risk factors on the risk of recurrence remains unclear. Although some studies have indicated a “slightly increased risk of recurrent VTE” (12), others have reported negative results or even a lower risk in older patients (6, 10, 11, 13). Patients with provoked VTE reportedly tend to have a low risk of VTE recurrence (10, 14), while those with unprovoked VTE have a high risk (10) or intermediate risk when therapy is stopped (8).

Though the methods of diagnosis and treatment of VTE are largely agreed upon, it is not known how they are applied in everyday practice. Moreover, the epidemiology of VTE has not been adequately described in Saudi Arabia. Therefore, the main aim of this study was to assess the clinical characteristics, risk factors, and diagnostic methods of patients with VTE in Saudi Arabia. We also aimed to obtain a realistic overview of the management and clinical outcomes of patients with VTE.

## Materials and methods

### Study design and setting

This was a retrospective registry-based single center study of VTE patients at King Fahad Medical City (KFMC), Riyadh, Saudi Arabia, over ten years.

### Study participants

All adult (age > 14 years) inpatients and outpatients referred to the thrombosis unit of the KFMC with clinically suspected VTE were eligible for the study.

### Inclusion criteria

All inpatients and outpatients confirmed with DVT or PE or both by objective test, and age > 14-year-old.

### Exclusion criteria

Patients with superficial vein thrombosis or thrombophlebitis, unusual site thromboses such as splanchnic vein thrombosis (i.e., thrombosis in the mesenteric, splenic or portal veins), retinal vein thrombosis and cerebral vein thrombosis, patients without an objective diagnosis of VTE, arterial thrombosis such as stroke, acute myocardial infection or peripheral arterial disease, recurrent abortion, pregnancy complications, and VTE prophylaxis.

### Diagnostic criteria

The diagnosis of DVT was established by compression ultrasonography and PE was established by computerized tomography scan or ventilation perfusion scan.

### Data collection

Data were collected using a standardized case report form that included the following information: demographic and clinical characteristics, risk factors, diagnostic methods, management, and outcome. Demographic and clinical characteristics included the age at diagnosis; sex; marital status; body mass index (BMI) category; previous history of VTE; family history of VTE; history of hypertension, diabetes, smoking, peripheral vascular disease, varicose veins, immobilization of the affected limb, and recent hospitalization within 3 months for a medical condition; and the site of VTE. Trauma, pregnancy, long travel, hospitalization, surgery, cancer, and oral contraceptive pills (OCP) were identified as risk factors for provoked VTE.

With respect to VTE management, the following data were collected: thrombolytic therapy, catheter-directed thrombolysis, initiation of anticoagulation therapy, maintenance therapy, placement of inferior vena cava filter (IVC), and duration of anticoagulation.

The outcomes of the current VTE episode included death, discharge after improvement, hospitalization, and admission to the intensive care unit (ICU).

### Ethical considerations

The study was approved by the institutional review board of KFMC. The requirement for informed consent was waived owing to the retrospective study design.

### Sample size

A recent study reported that the prevalence of unprovoked VTE was ~60% (1). Considering the baseline prevalence of unprovoked VTE as 60%, a power of 90, and a 95% confidence interval with a 5% margin of error, the minimum required sample size was 804.

The sample size was calculated by using OpenEpi software and the following formula


(1) $$\begin{array}{r} n=\frac{Z^{2*}P\left(1-P\right)}{d^{2}} \end{array}$$

Where n = sample size.

Z = level of confidence (2 sided 95% confidence interval).

*P* = prevalence of unprovoked VTE.

*d* = margin of error.

### Statistical analysis

Data were analyzed using SPSS 24.0 (IBM Corp., Armonk, NY, USA). Age is presented as the median and interquartile range (IQR). Categorical variables are presented as counts and percentages. The chi-square test and Fisher's exact test were used to assess the association between two categorical variables. Multivariable logistic regression analysis was performed to assess potential risk factors for VTE recurrence. Statistical significance was set at *p* < 5%.

## Results

### Baseline characteristics

**Figure 1:**
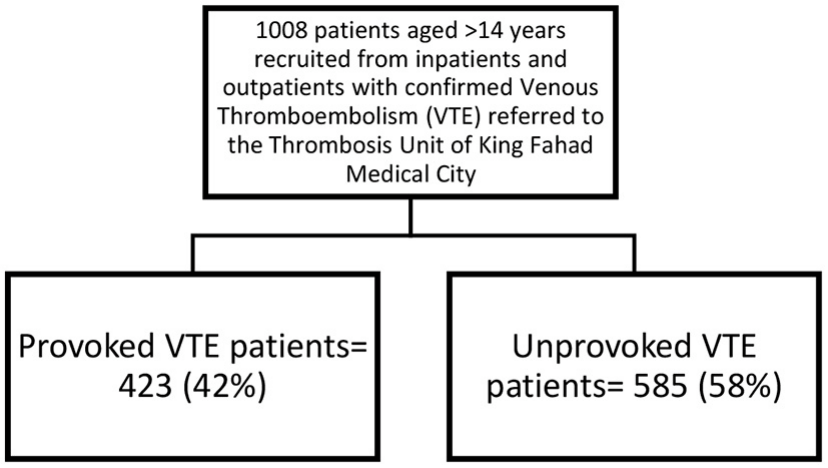
Flowchart of selected patients and classified according to provoking status.

A total of 1,008 patients were recruited. The median age at diagnosis was 40 years (IQR: 65 years). Six hundred patients (61%) were affected by DVT, 285 patients (28.2%) by PE and 108 patients (10.8%) by both DVT and PE. Most patients were female (*n* = 738, 73.2%), Saudi (*n* = 973, 96.5%), married (*n* = 850, 92.9%), and obese (*n* = 463, 46.1%). More than half of the patients had unprovoked VTE (*n* = 585, 58%), where 423 (42%) had provoked VTE (Table 1, Figure 1).

**Table 1:** Demographic characteristics of the study participants.

**Characteristics**	***n***	**%**
**Gender, *n* (%)**		
Male	270	26.8
Female	738	73.2
**Marital status, *n* (%)**		
Married	850	92.9
Not married	128	7.1
**Nationality, *n* (%)**		
Saudi	937	96.5
Non-Saudi	71	3.5
**Body mass index (BMI), *n* (%)**		
Underweight	17	1.7
Normal	158	15.7
Overweight	366	36.5
Obese	463	46.1
**Provoking status**		
Yes	423	42
No	585	58
Age at diagnosis, Median (IQR)	40 (65)	

Table 2 shows the distribution of provoked and unprovoked VTE according to demographics and risk factors. The results suggest that risk factors for provoked VTE and unprovoked VTE were more common among women, married, obese, those with no history of VTE, no family history of VTE, no hypertension, no diabetes, non-smokers, and with no history of recent hospitalization for a medical condition within the previous 3 months. Additionally, almost none of the patients had peripheral vascular disease, varicose veins, or immobilization of the affected limb. There was a statistically significant association between provoked status and sex, family history of VTE, smoking, underlying peripheral vascular disease or varicose veins, recent hospitalization within 3 months for a medical condition, and the site of VTE (*p* < 0.05). The median age of the patients at diagnosis was somewhat comparable between the two groups (provoked vs. unprovoked VTE, *p* = 0.235). In each patient group, the most common VTE event was deep vein thrombosis (DVT) alone, with the highest percentage of DVT events occurring in patients with provoking factors (69.7%) (Table 2).

**Table 2:** Risk factors in patients with provoked vs. unprovoked venous thromboembolism.

**Characteristic**	**Provoked VTE (*****n*** = **423)**	**Unprovoked VTE** **(*****n*** = **585)**	***p*** **-value**
Age at diagnosis (Median, IQR)	39 (20)	40 (22)	0.235
**Gender, *n* (%)**			
Male	75 (17.7)	195 (33.3)	<0.001
Female	348 (82.3)	390 (66.7)	
**Marital status, *n* (%)**			
Married	380 (94.5)	470 (91.6)	0.089
Not married	22 (5.5)	43 (8.4)	
**BMI categories, *n* (%)**			
Underweight	9 (2.1)	8 (1.4)	0.073
Normal	77 (18.2)	81 (13.9)	
Overweight	142 (33.6)	224 (38.6)	
Obese	195 (46.1)	268 (46.1)	
**Previous history of VTE, *n* (%)**			
Yes	88 (21.3)	158 (27.3)	0.069
No	326 (78.7)	420 (72.7)	
**Family history of VTE, *n* (%)**			
Yes	39 (11.2)	22 (5.0)	0.001
No	310 (88.8)	421 (95.0)	
**History of hypertension, *n* (%)**			
Yes	83 (19.8)	133 (23.3)	0.271
No	326 (80.2)	439 (76.7)	
**History of diabetes, *n* (%)**			
Yes	87 (19.8)	116 (20.2)	0.856
No	333 (80.2)	457 (79.8)	
**Smoking, *n* (%)**			
Yes	5 (1.2)	29 (5.1)	<0.001
No	414 (98.8)	543 (94.9)	
**Peripheral vascular disease*, n* (%)**			
Yes	6 (1.4)	20 (3.5)	0.045
No	412 (98.6)	552 (96.5)	
**Varicose veins, *n* (%)**			
Yes	3 (0.7)	14 (2.4)	0.039
No	415 (99.3)	558 (97.6)	
**Immobilization of the affected limb, *n* (%)**			
Yes	14 (3.8)	13 (2.5)	0.271
No	353 (96.2)	503 (97.5)	
**Recent hospitalization within 3 months for a medical condition, *n* (%)**			
Yes	25 (6.7)	17 (3.3)	0.018
No	349 (93.3)	502 (96.7)	
**Site of VTE**			
DVT only	295 (69.7)	320 (54.7)	<0.001
PE only	85 (20.1)	200 (34.2)	
DVT and PE	43 (10.2)	65 (5.9)	

*Percentages sum to different numerators because of missing values. The bold values indicate the significant P values*.

**Figure 2:**
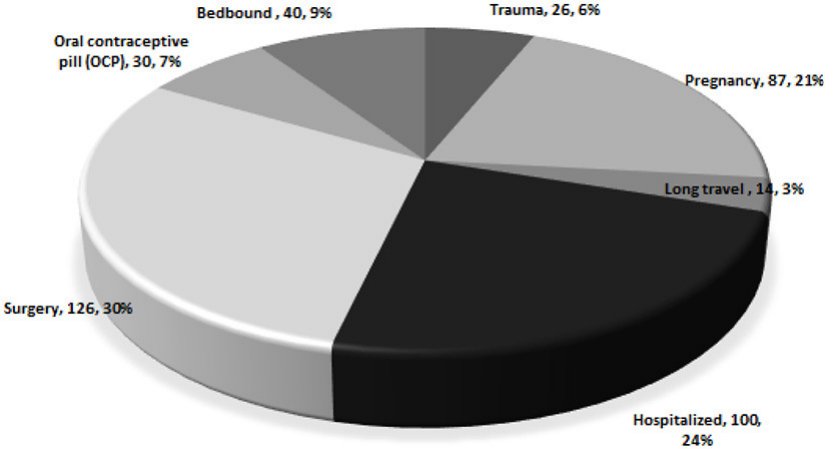
Distribution of causes of provoked VTE (*n* = 423).

Figure 2 shows the distribution of the causes of provoked VTE. The most common causes of death were surgery (*n* = 126, 29.8%), hospitalization (*n* = 100, 24.2%), and pregnancy (*n* = 87, 20.6%). Other causes were also reported, including being bedbound (*n* = 40, 9.5%), use of OCP (*n* = 30, 7.1%), underlying trauma (*n* = 26, 6.1%), and history of long travel (*n* = 10, 3.3%).

### VTE management features and outcome

The majority of patients with DVT needed hospitalization (*n* = 543, 88.3%), in most cases, for ≤3 days (*n* = 433, 79.9%), while less than one-half of PE patients needed hospitalization (*n* = 129, 45.3%), with > 10 days being the most frequent period (*n* = 44, 34.1%). Furthermore, most patients showed improvement and were discharged in both DVT (*n* = 385, 70.9%) and PE (*n* = 88, 68.2%) groups. However, a few in each group required admission to the ICU (DVT, *n* = 9; PE, *n* = 7). With respect to outcomes in the ICU, four of nine DVT patients improved and were discharged (45.5%), while five out of seven PE patients improved and were discharged (71.4%) (Table 3).

**Table 3:** Management and outcome of the current episode of venous thromboembolism.

	**DVT**	**PE**
**Hospitalized**	*n* (%)	*n* (%)
Yes	543 (88.3)	270 (94.7)
No	72 (11.7)	15 (5.3)
**Duration of hospitalization**		
≤ 3 days	433 (79.7)	38 (29.5)
4–7 days	44 (8.1)	23 (17.8)
8–10 days	20 (3.7)	24 (18.6)
>10 days	46 (8.5)	44 (34.1)
**Outcome**		
Died	158 (29.1)	41 (31.8)
Improved and discharged	385 (70.9)	88 (68.2)
**Admission to ICU**		
Yes	9 (1.7)	7 (5.4)
No	534 (98.3)	122 (94.6)
**ICU outcome**		
Died	5 (55.5)	2 (28.6)
Improved and discharged	4 (44.5)	5 (71.4)

As for treatment modalities, thrombolytic therapy was administered in 14.1% (*n* = 142) of patients, while catheter-directed thrombolysis was used in 1.0% (*n* = 10). Patients were also administered anticoagulation therapy followed by maintenance therapy. The most common anticoagulation therapy was low-molecular-weight heparin LMWH (*n* = 591, 58.1%). This was followed by factor Xa inhibitors such as rivaroxaban (*n* = 407, 40.0%) and heparin infusion (*n* = 19, 1.9%) (Table 4).

**Table 4:** Treatment of patients with venous thromboembolism.

**Treatment**	***N***	**%**
**Thrombolytic therapy**		
Yes	142	14.1
No	866	86.3
**Catheter-directed thrombolysis**		
Yes	10	1.0
No	998	99.0
**Initiation anticoagulation therapy**		
Low-molecular-weight heparin (LMWH )	591	58.1
Heparin infusion	19	1.9
Factor Xa inhibitors(Rivaroxiban)	407	40.0
**Maintenance therapy**		
Vitamin-K antagonist (Warfarin)	433	43.0
Factor Xa inhibitors (Rivaroxiban)	543	56.0
Direct thrombin inhibitors (Dabigatran)	10	1.0
**Inferior vena cava filter (IVC)**		
Yes	15	1.5
No	913	93.7
Do not know	46	4.7
**Duration of anticoagulation**		
3–6 months	544	54.0
Extended duration	464	46.0

Following initial therapy, maintenance therapy was administered. The majority of patients were administered the Vitamin-K antagonist (VKA), warfarin (*n* = 433, 43.0%), followed by factor Xa inhibitors, such as rivaroxaban (*n* = 543, 56.0%) and direct thrombin inhibitors, such as dabigatran (*n* = 10, 1.0%) (Table 4).

Few participants underwent IVC filter placement for known significant PE, DVT, or both (*n* = 15, 1.5%). More than half of the patients had received anticoagulation therapy for VTE for 6 months (*n* = 544, 54.0%), while others had been prescribed anticoagulation for an extended duration (*n* = 464, 46.0%) (Table 4).

Table 5 shows the unadjusted odds ratio of clinical outcomes for provoked and unprovoked VTE. The odds of mortality with provoked VTE were 3.20 times higher than those of mortality with unprovoked VTE = [OR = 3.20, (2.12–4.83; *p*-value < 0.001)]. Moreover, the odds of VTE recurrence were 81% lower in patients with provoked VTE than in those with unprovoked VTE [OR = 0.19, (0.12–0.30; *p* < 0.001)].The odds of having major bleeding were 2.56 times higher in patients with provoked VTE than in patients with unprovoked VTE [OR = 2.56, (1.29–5.04; *p*-value = 0.007)].

**Table 5:** Unadjusted odds ratios of clinical outcomes for provoked vs. unprovoked venous thromboembolism.

**Outcome**	**Provoked VTE**	**Unprovoked VTE**	**OR (95% CI)**	***p*** **-value**
Death	118 (48.0)	81 (30.1)	3.20 [2.12–4.83]	<0.001
Recurrence VTE	35 (8.2)	122 (22.3)	0.19 [0.12–0.30]	<0.001
Major bleeding	27 (6.4)	14 (2.4)	2.56 [1.29–5.04]	0.007

*Values of provoked VTE and unprovoked VTE are n/N (%), OR, Odds ratio; CI, confidence interval*.

We also investigated the relationship between anticoagulant therapy duration and VTE recurrence outcomes. Patients were categorized into two groups: the first included those with no prescription of VKA or a treatment duration of 3–6 months, and the second group comprised patients who received VKA treatment for an extended duration (i.e., more than 6 months). Less than one-fifth of patients (*n* = 155, 18.3%) had received VKA anticoagulation treatment for 3–6 months, while 81.7% of patients received an extended duration of VKA (Group II, *n* = 691). These patients tended to be 2.88 times more likely to experience VTE recurrence than those who received VKA anticoagulation treatment for 3–6 months [OR = 2.88, 95% CI: (1.58–5.22)]. Moreover, those who were prescribed an extended duration of VKA anticoagulation treatment had a 3.27-fold mortality risk [OR = 0.44, 95% CI: (0.29–0.67)] compared to those who were prescribed VKA anticoagulation treatment for 3–6 months [OR = 3.27, 95% CI: (1.9–5.59)]. Duration of anticoagulation was not significantly associated with a major bleeding outcome and the risk of major bleeding was similar between the two groups [OR = 4.05, 95% CI: (0.96–17.04)] (Table 6).

**Table 6:** Comparison between duration of Vitamin-K antagonist therapy.

	**Duration of VKA anticoagulation**			
**Outcome**	**Group I**	**Group II**		
**VTE recurrence**	***n* (%)**	***n* (%)**	**OR [95% CI]**	***p*-value**
Yes	13 (8.4)	144 (20.8)	2.88 [1.58–5.22]	<0.001
No	142 (91.6)	547 (79.2)		
**Death**
Yes	19 (19.0)	175 (43.4)	3.27 [1.91–5.59]	<0.001
No	81(81.0)	228 (56.6)		
**Major bleeding**
Yes	2 (1.5)	36 (6.0)	4.05 [0.96–17.04]	0.056
No	128 (98.5)	569 (94.0)		

*Group I, includes subjects with no prescription of VKA, or treatment duration of 3–6 months; Group II, includes patients who received VKA treatment for an extended duration*.

### Risk factors for VTE recurrence

A total of 157 of 1,008 (15.6%) patients experienced VTE recurrence during the study period. The results of the multivariate logistic regression analysis assessing the risk factors for VTE recurrence are shown in Table 7. The model showed that 86.2% of patients were correctly classified. Higher odds of VTE recurrence were associated with older age [OR = 2.3, CI (1.1–4.7)]. Males were 3.4 times more likely to have recurrent VTE than women [OR = 3.4, CI (2.1–5.7)]. Moreover, obesity was a significant risk factor for VTE recurrence over underweight, though not over normal and overweight patients. That is, the odds of VTE recurrence in obese patients were 2.7 times those who were underweight [OR = 2.6, CI (1.3–4.8)]. A prior history of VTE and a family history of VTE were found to be associated with higher odds of VTE recurrence {5.6 and 3.8 times higher odds than those without prior history of VTE [OR = 5.6, CI (2.8–12.4)] and without family history of VTE [OR = 3.8, CI (1.8–7.5)], respectively}. With respect to the site of VTE, those with either DVT [OR = 2.2, CI (1.4–4.9)] or PE [OR = 3.1, CI (1.6–8.5)] had higher odds of VTE recurrence than those with both DVT and PE.

**Table 7:** Results of multivariate logistic regression analyzing the risk factors for recurrence of venous thromboembolism.

**Risk factors**	**Odds ratio**	**Confidence**
	**(OR)**	**interval (CI)**
Age	2.3	1.1–4.7
**Gender**	
Male	3.4	2.1–5.7
Female, reference	1	Referent
**BMI categories**	
Underweight, reference	1	Referent
Normal	1.1	0.7–1.9
Overweight	1.2	0.9–2.1
Obese	2.6	1.3–4.8
**Previous history of VTE**	
Yes	5.6	2.8–12.4
No	1	Referent
**Family history of VTE**	
Yes	3.8	1.8–7.5
No	1	Referent
**Type of VTE**	
DVT only	2.2	1.4–4.9
PE only	3.1	1.6–8.5
DVT and PE	1	Referent
**Provoking status**	
Unprovoked	1.28	0.8–2.6
Provoked	1	Referent
**Causes of provoked VTE**	
Trauma	1.8	1.2–4.3
Pregnancy	3.7	1.7–8.6
Hospitalized	2.1	1.5–6.4
Surgery	2.3	1.5–7.6
Oral contraceptive pill (OCP)	0.97	0.7–2.3
Bedbound	1.1	0.8–3.2
Long travel, reference	1	Referent
**Duration of anticoagulation**	
3–6 months	3.7	1.7–6.3
Extended duration	1	Referent

Furthermore, the cause of VTE was found to be a significant risk factor for recurrence. The odds of VTE recurrence in pregnant women were 3.7 times higher than that in those with a history of long travel [OR = 3.7, CI (1.7–8.6)]. Moreover, patients who underwent recent surgery had 2.3-fold higher odds of VTE recurrence than those who traveled for a long time [OR =2.3, CI (1.5, 7.6)]. The odds of recurrent VTE were 2.1-fold greater in hospitalized patients than in those who had traveled for a long time [OR = 2.1, CI (1.5–6.4)]. Patients with trauma were 1.8 times more likely to have VTE recurrence than those who had traveled for a long time [OR = 1.8, CI (1.2–4.3)]. Nevertheless, the odds of VTE recurrence in patients who took OCP [OR = 0.97, CI (0.7, 2.3)] and were bedbound [OR = 1.1, CI (0.8, 3.2)] were similar to those in patients who had traveled long periods. As for the duration of anticoagulation treatments, patients who were prescribed anticoagulation treatment for 3–6 months had 3.7-fold higher odds of recurrence of VTE than those who were prescribed anticoagulation treatment for an extended duration [OR = 3.7, CI (1.7, 6.3)]. Provoked and unprovoked patients had similar odds of experiencing recurrence [OR = 1.28, CI (0.8, 2.6)].

## Discussion

In our registry, more than half of the patients (58%) had unprovoked VTE, with the majority being diagnosed with DVT alone (61.0%). Also, both provoked and unprovoked VTE were more common among women, married, obese, and patients with DVT. However, the median age was comparable between the two groups. A recent meta-analysis showed that the incidence of unprovoked VTE was higher in men and older patients than in those with provoked VTE (15). This can be reasoned through the possible increase in coagulability with age (16). Potential explanations for the higher incidence rate of VTE among women include the higher proportion of women enrolled in the current study, especially those with provoked VTE, and the possibility of estrogen use, pregnancy, or OCP use among women. DVT was the most prevalent type of VTE among all patients; however, it was the most frequent in patients with provoked VTE. PE, meanwhile was higher in patients with unprovoked VTE than in those with provoked VTE. The results of the current study are somewhat congruent with those of studies performed elsewhere (17–21). For example, a recent study by Ageno et al. (17) used the Global Anticoagulant Registry in the FIELD (GARFIELD)-VTE, consisting of 10,207 patients in 28 countries, and reported that 59.2% of patients had unprovoked VTE, and DVT was the most common site of VTE in both provoked and unprovoked VTE groups. Moreover, patients with transiently provoked VTE were more likely to be younger and female compared to those with unprovoked VTE (17). The Italian MASTER VTE registry reported that 72.7% of patients were diagnosed with DVT, 9.7% with PE, and 17.5% with both DVT and PE in Italy during the period from 2002 to 2004 (18). In Korea, the Korean VTE registry indicated that about 72% of patients had provoked VTE, and the main risk factors for VTE were older age, female sex, cancer, immobilization, surgery, severe medical disease, stroke, and trauma. Moreover, the prevalence of PE was higher in patients with unprovoked VTE than in those with risk factors for provoked VTE (19), similar to the findings of this study. Cohen et al. (20) used the Prevention of thromboembolic Events-European Registry in Venous Thromboembolism (PREFER in VTE) and found that DVT was diagnosed in 59.5% of patients, while PE was seen in 40.5%; also, more than half of the patients were male (52.0%) and older, dissimilar to our study. In Japan, Nakamura et al. (21) used the Japan VTE Treatment Registry (JAVA) to address VTE management and outcomes, and found that 68.7% of were diagnosed with isolated DVT, 17.0% with PE, and 14.4% with both DVT and PE; 43.2% of patients had unprovoked VTE (e.g., idiopathic). They also showed that a family history of recent surgery, history of VTE, and medical history of cancer were the most common risk factors for VTE (21).

The findings of this study revealed that the most frequent causes of provoked VTE were surgery, hospitalization, and pregnancy, followed by bedbound status, OCP use, trauma, and long travel. These findings are consistent with the recently reported VTE-related literature that might influence the duration of treatment and prognosis (8, 21–26). Moreover, most patients in our study were treated at hospitals. Thrombolytic therapy was used in 14.1% of patients with acute VTE, and catheter-directed thrombolysis was used in 1.0% of patients. This observation is similar to that of a recent study conducted in Japan (21). Furthermore, patients were usually treated with an initial therapy of LMWH and rivaroxaban, which is congruent with the current guidelines of the American Society of Hematology (ASH) for optimal management of anticoagulation therapy in patients with VTE (27), as well as some previous studies (17, 28). The proportion of hospitalized patients diagnosed with DVT was higher than that of patients diagnosed with PE. The mean duration of hospitalization was, however, higher in patients with PE (10 days) than in those with DVT (3 days or lower). Most patients improved and were discharged in both the DVT and PE groups, but a few of them required admission to the ICU. A recent study indicated that the mean length of hospital stay for PE was 5.2 days compared to an average of 4.7 days for DVT patients, which is not consistent with our report (29).

Furthermore, our study showed that patients were prescribed anticoagulation treatment following initial therapy–more than half of the patients were prescribed rivaroxaban. The VKA, warfarin, was prescribed in 43.0% of patients, and direct thrombin inhibitors such as dabigatran were prescribed in 1.0% of patients. More than half of the patients were given anticoagulation therapy for 3–6 months, and an appropriate international normalization ratio (INR) was correctly targeted for, contradicting the ASH guidelines (27). In the GARFIELD-VTE registry, more than half of the patients were prescribed direct oral anticoagulants, while about one-thirds were on warfarin on the first day of treatment, which is not consistent with this study (17, 28). Nakamura et al. (21) stated that VKA was used in 88.8% of patients in Japan, with a mean duration of 7 months. Although the American College of Chest Physicians recommends prescribing VKA for 3 months after a provoked VTE diagnosis and for more than 3 months if it is caused by active cancer, studies have shown that more than 40% of participants remained on VKA therapy for 12 months (17, 30, 31). Very few patients in this study received IVC for known significant PE, DVT, or both. This observation was similar to that by Muriel et al. (32) who found that IVC was used in 371 of 40,142 patients, but was not consistent with that by Nakamura et al. (21) who reported that approximately 40.7% of patients underwent IVC placement.

As for the treatment outcomes over the study period, patients with VTE were at an increased risk of mortality and major bleeding than those with unprovoked VTE. Conversely, patients with unprovoked VTE were at an increased risk of VTE recurrence compared to those with provoked VTE. This result is not consistent with Ageno et al. (17) who showed that differences in VTE recurrence rates were not evident between provoked and unprovoked VTE patients after adjusting for patient characteristics, which is not consistent with our findings, and that patients with risk factors for provoked VTE had increased risks of mortality and major bleeding compared to those with unprovoked VTE, which confirms our findings. Recent evidence also suggests that patients with minor provoking factors are at increased risk of recurrent VTE, and thus require long-term anticoagulation, despite long-term anticoagulation generally being needed for unprovoked VTE, suggesting a greater concern for VTE recurrence than for major bleeding (33). Previous research has also shown that provoked VTE patients with minor risk factors have an increased risk of recurrent VTE compared to those with major risk factors (8, 21, 22, 24). Concerning treatment duration, the results of this study indicated that patients prescribed anticoagulation treatment for an extended duration were at an increased risk of recurrent VTE and mortality than those treated for 3–6 months; however, this was not the case for major bleeding. An extended duration of VKA treatment for more than 6 months was associated with an increased risk of mortality and recurrent VTE. Nevertheless, the risk of major bleeding was comparable in both VKA treatment groups, which is inconsistent with the results obtained by Nakamura et al. (21) in the JAVA study. Long-term and extended anticoagulation duration may play a vital role in the overall prognosis of VTE. In the JAVA study, 76 patients did not benefit from warfarin, who had about five-times higher mortality rate than those who received VKA therapy, which might be attributed to confounding factors such as underlying comorbidities that prompted the decision not to treat (21). The MASTER registry indicated that the lack of anticoagulation was significantly associated with a 3.2-fold increase in the mortality rate (34).

A total of 157 cases of VTE recurrence were reported in this study. The results of the multivariable logistic regression showed that older age, male sex, obesity, family history of VTE, history of VTE, type of VTE, pregnancy, hospitalization, surgery, trauma, and 3–6 months duration of anticoagulation treatment were associated with greater odds of recurrence. Different studies have shown somewhat comparable results, while others have contradicted them (14, 17, 21, 24, 26, 32, 35–42). For instance, the recurrence rate in PE patients was two times higher than in those with DVT, and the rates of VTE recurrence were higher in cancer patients with lower anticoagulation duration; however, this rate was similar among patients regardless of sex, presence of cancer, body mass index, previous VTE, IVC filter, and warfarin, which is not consistent with the current study (21). In contrast, Louzada et al. performed a retrospective study and reported that women, underlying lung cancer, and a history of previous VTE were associated with higher VTE recurrence risks. However, patients with breast and localized cancers had a lower risk of VTE recurrence (39). A meta-analysis showed that men were associated with a 50% higher risk of VTE recurrence than women, regardless of provoking status (41). Zhu et al. (14) reviewed a wide range of studies on risk factors for recurrent VTE and showed that an increased risk of recurrent VTE was present in patients with persistent risk factors such as cancer, while the risk was lower in patients with transient provoking factors, normal D-dimer levels, and the absence of residual venous thrombosis after discontinuation of oral anticoagulation. Therefore, inconsistencies in previous studies may be attributed to differences in the study design and patient selection.

The present study has numerous limitations. First, details of precise VTE management, such as the duration of anticoagulation, was not appropriately recorded, leading to missing data. The data were also obtained from only one site, which might not reflect the general VTE population in Saudi Arabia. The lack of appropriate variables to record patients' treatment preferences is an additional limitation. Furthermore, we were not able to perform survival analyses, owing to restrictions with the currently available data. Nevertheless, the strengths of the present study include its power to determine the distribution of VTE risk factors, including provoked and unprovoked VTE, and its ability to provide more detailed outcomes, including recurrence of VTE, mortality, and major bleeding.

## Conclusion

This study showed that unprovoked VTE was, to some extent, more prevalent than provoked VTE among a cohort of patients in Saudi Arabia, suggesting genetic susceptibility. The most important risk factors for VTE recurrence were male sex, family history of VTE, prior history of VTE, type of VTE (specifically, both DVT and PE), obesity, trauma, surgery, hospitalization, surgery, pregnancy, and 3–6 months of anticoagulation treatment. The treatment patterns and clinical outcomes outlined in this study were somewhat comparable with the current literature, thus improving our understanding of the epidemiology and risk factors of VTE and recurrent VTE in the Saudi cohort. Therefore, deep consideration of the current findings can allow for the optimal use of prophylactic approaches and anticoagulation treatment, and improve clinical outcomes of VTE in practice.

## Data availability statement

The raw data supporting the conclusions of this article will be made available by the authors, without undue reservation.

## Ethics statement

The studies involving human participants were reviewed and approved by KFMC-IRB. Written informed consent for participation was not required for this study in accordance with the national legislation and the institutional requirements.

## Author contributions

MA carried out the study, participated in the study design, and wrote the final manuscript. FA conceived of the study and participated in its design and in drafting the manuscript. MKA and AA-S participated in the study design, interpretation of data, and drafting of the manuscript. AGA contributed to the design of the study, managed the literature search, and drafted the manuscript. AFA participated in the interpretation of data and drafting the article. All authors read and approved the manuscript.

## Conflict of interest

The authors declare that the research was conducted in the absence of any commercial or financial relationships that could be construed as a potential conflict of interest.

## Publisher's note

All claims expressed in this article are solely those of the authors and do not necessarily represent those of their affiliated organizations, or those of the publisher, the editors and the reviewers. Any product that may be evaluated in this article, or claim that may be made by its manufacturer, is not guaranteed or endorsed by the publisher.
